# Influence of pH on the Morphology and Cell Volume of Microscopic Algae, Widely Distributed in Terrestrial Ecosystems

**DOI:** 10.3390/plants13030357

**Published:** 2024-01-25

**Authors:** Lira A. Gaysina

**Affiliations:** 1 Department of Bioecology and Biological Education, M. Akmullah Bashkir State Pedagogical University, 450008 Ufa, Russia; lira.gaisina@gmail.com; 2 All-Russian Research Institute of Phytopathology, 143050 Bolshye Vyazemy, Russia

**Keywords:** adaptation, authentic strains, cell wall, discoloration, protoplast, tolerance, *Bracteacoccus minor*, *Chlorella vulgaris*, *Chlorococcum infusionum*, *Pseudococcomyxa simples*, *Vischeria magna*

## Abstract

Terrestrial algae are a group of photosynthetic organisms that can survive in extreme conditions. pH is one of the most important factors influencing the distribution of algae in both aquatic and terrestrial ecosystems. The impact of different pH levels on the cell volume and other morphological characteristics of authentic and reference strains of *Chlorella vulgaris*, *Bracteacoccus minor*, *Pseudoccomyxa simplex*, *Chlorococcum infusionum*, and *Vischeria magna* were studied. *Chlorella vulgaris*, *Pseudoccomyxa simplex,* and *Vischeria magna* were the most resistant species, retaining their morphology in the range of pH 4–11.5 and pH 3.5–11, respectively. The change in pH towards acidic and alkaline levels caused an increase in the volume of *Pseudoccomixa simplex* and *Vischeria magna* cells, according to a polynomial regression model. The volume of *Chlorella vulgaris* cells increased from a low to high pH according to a linear regression model. Changes in pH levels did not have a significant impact on the volume of *Bracteacoccus minor* and *Chlorococcum infusionum* cells. Low and high levels of pH caused an increase in oil-containing substances in *Vischeria magna* and *Bracteacoccus minor* cells. Our study revealed a high resistance of the studied species to extreme pH levels, which allows for us to recommend these strains for broader use in biotechnology and conservation studies of natural populations.

## 1. Introduction

The concentration of hydrogen ions plays a fundamental role in determining the boundaries of the existence of living matter. Most living organisms exist at a pH ranging from 4 to 9. The limiting concentration of hydrogen ions, above and below which currently known organisms stop growing and multiplying, fluctuates within a pH range from 1 to 11 [1]. If the pH value does not approach its extreme values, communities can regulate changes in this factor by changing the intensity of respiration and the functioning of the body’s enzyme systems [2].

There are numerous data in the literature concerning the effects of pH on various groups of organisms, including algae. Algae have a wide-ranging tolerance to pH, but the limits of resistance to this factor in representatives of various systematic groups are not the same [3,4,5]. Through examining the influence of pH on the rate of vegetative division [6], zoospores’ differentiation [7], the amount of available iron in the medium [8,9], the ratio of nitrogen and carbon, autotrophic and heterotrophic nutrition [10,11,12,13,14,15], proteins, lipids, carbohydrates, fatty acids, chlorophyll content and biomass [16,17], algae assemblages in water and terrestrial ecosystems [18,19,20,21,22], and algal cell membrane permeability [23], growth [24,25], toxicity [25], morphology [26], and physiology [27] have been established. 

In recent decades, due to the development of algae biotechnology, numerous studies have estimated the impact of various factors on the effectiveness of cultivation. These studies have shown that pH is one of the main factors influencing the growth of algae in photobioreactors and open ponds [28,29,30,31,32,33,34]. In addition to pH, the nitrogen concentration, gas flow rate, and light intensity influenced the carbon dioxide sequestration during algae cultivation [35].

It should be noted that the vast majority of algological studies (including environmental ones) are devoted to the investigation of aquatic algae, while terrestrial forms remain extremely insufficiently studied. At the same time, it is well known that terrestrial algae are highly resistant to extreme levels of ecological factors. This fact allows for them to be used as a model biota for studying the mechanisms of resistance of living organisms [36], including extreme pH values. 

For this reason, the study of the autecology of the most widespread species, which are detected in various habitats around the world, can contribute to our growing knowledge of their adaptations to varying environmental conditions. Among the species of high importance are *Bracteacoccus minor* (Schmidle ex Chodat) Petrová, *Chlorococcum infusionum* (Schrank) Meneghini, *Chlorella vulgaris* Beijerinck, *Pseudococcomyxa simplex* (Mainx) Fott, and *Vischeria magna* (J. B. Petersen) Kryvenda, Rybalka, Wolf, and Friedl. 

*Bracteacoccus minor* is widely distributed in the terrestrial habits [37,38,39,40] and belongs to the cosmopolitans [41]. In addition, this species is often found in extreme habitats. For example, it is widely distributed in spruce forests that have been exposed to acid rain in the Czech Republic [42]. 

*Chlorococcum infusionum* is a type species of the genus *Chlorococcum* [43]. *Chlorococcum infusionum* has been found in both soil and stagnant water bodies [37]. It is assumed that this species belongs to cosmopolitans, it is found both in neutral and acidic soils, as well as on porous acid rocks [44]. *Chlorococcum infusionum* is common in all zones in almost all types of soils, from waterlogged areas to high-altitude deserts, and from acidic podzolic to highly saline carbonate [45,46,47,48]. This species has been detected in polluted urban soils [49]. *Chlorococcum infusionum* has a high indicator value for the soils of spruce forests in the background areas of the middle and southern taiga [50]. 

*Chlorella vulgaris* is a type species of the genus *Chlorella* [43]. This species is common in reservoirs, soils, and terrestrial substrates of various types, and it belongs to the cosmopolitans [37,38,41,48,51]. *Chlorella vulgaris* is widely distributed in soils and subaerial habitats. This species has a large number of local physiological and ecological races living in different conditions [44]. *Chlorella vulgaris* has been identified in salt marshes, as well as in acidic and polluted soils [52]. This species remains the only taxon in conditions of severe oil pollution [53] and severe acidification during precipitation near metallurgical enterprises [52]. However, it should be noted that most of the findings regarding this species need to be verified, since it is quite common practice to assign any small green spherical algae to *Chlorella vulgaris* [44].

*Pseudococcomyxa simplex* is a type species of the genus *Pseudococcomyxa* [43]. *Pseudococcomyxa simplex* is one of the widespread species of terrestrial algae [38,41,46,47,54]. It has a wide ecological plasticity; for example, these algae are highly resistant to a low pH. The species is found in very acidic soil with a pH < 3 in Italy [42]. In addition, *Pseudococcomyxa simplex* was found in all the studied sites during an investigation of spruce forest algae exposed to acid rain in Northern Bohemia (Czech Republic) [42]. 

*Vischeria magna* has a wide distribution around the world; it has been found in North America [55,56], Europe [38,42,47], Africa [39] and Asia [50]. This species is also found in territories with extreme habitat conditions, for example, different types of salt marshes. *Vischeria magna* has been detected in the crustal salt flats in the chernozem, chestnut, and brown zones; in the middle salt flats in the chernozem and chestnut zones; in the deep salt flats in the chernozem, chestnut, brown zones; in the rejuvenated salt flats in the chernozem zone; and in the meadow salt flats in the chernozem zone [57]. Furthermore, this alga has been found in the acid rainfall zones in spruce forests in the Czech Republic and Germany [42]. 

An analysis of the literature data on the influence of ecological factors on microscopic algae revealed two main groups of criteria, which were used to assess the degree of effect:

1. Morphological criteria: cell size and shape [26,58], cell envelope ultrastructure [59], chloroplast morphology and coloration [58], cytoplasm, membrane and mitochondria structures [60], cell wall integrity [61];
2. Physiological criteria: growth reactions [62,63,64,65,66,67], photosynthesis intensity [68], carbohydrate and protein content [16], pigment composition and assimilation [69], chemical composition [17], chlorophyll “a” and “b” fluorescence [70], transmembrane electrochemical gradient [71], fatty acid production [72], cell division, and biological volume (cell volume) [58].

The term “biological volume” or “volume” is widely used to assess the functioning of aquatic forms of algae. It reflects both the morphological and physiological features of living organisms. It has been shown that picoplankton algae of very small sizes (<2 microns in diameter) have a larger surface area to volume ratio, which ensures the effective absorption of nutrients and photons [73]. This indicator is widely used to characterize the state of aquatic bacteria [74], dinoflagellates [75], and infusoria [76], as well as microscopic algae [26,77,78,79]. It has been established that biological volume plays a very important role in the ecology of algae. For example, in marine diatoms, cell volume is associated with metabolic rate [80,81], growth rate [82], photosynthetic capacity [80], respiration rate [83,84], and asexual reproduction [85]. In addition, the cell volume influences nutrient uptake [86], light absorption [87], and primary production in marine ecosystems [84,88]. A correlation between the evolution of the genome size and the volume of diatoms has been established [89]. It is stressed that that the cell volume of diatoms is an important component of the global carbon cycle.

Studies on nitrogen uptake depending on the size of micro- and macroalgae revealed that microalgae absorbed nitrogen per unit of biomass much faster at both high and low concentrations of the element in comparison with macroalgae. In addition, microalgae had a greater degree of affinity for nitrogen than macroalgae [90]. These differences in the absorption rates among small and large algae are usually attributed to size-specific processes depending on the relative surface area (SA: V). A regression analysis confirmed that size-dependent differences in kinetic parameters can be related to the relative surface area for a wide range of algae of different sizes.

Biological volume is closely related to the ratio of surface area to volume (S/V ratio). Under stress conditions, organisms attempt to reduce the surface area to volume ratio. The most advantageous aspect in this respect is the spherical shape of the cells [91]. Thus, it is not surprising that many terrestrial microscopic algae living in extreme environmental conditions have spherical shapes. In a study on the morphological diversity, evolutionary relatedness, and size constraints of freshwater algae and cyanobacteria, it was proposed that “the morphospace may serve as a proxy for an ecospace,” and, in future, this morphospace could be used to demonstrate the current ecological processes [92].

This study aimed to estimate the resistance of algae with a wide geographical distribution to pH and the influence of pH levels on the morphology and biological volume of these taxa. 

## 2. Results

The effects of pH on the cell morphology and volume had their own peculiarities and characteristics for each species, which are described below.

### 2.1. Bracteacoccus minor

**Figure 1:**
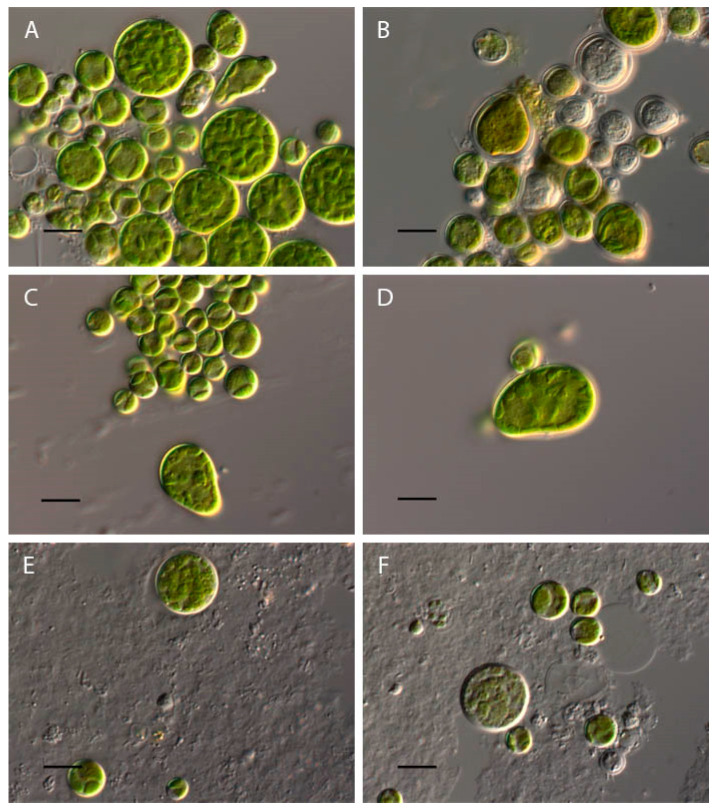
Influence of pH on *Bracteacoccus minor* morphology. (**A**) Control variant (pH 6.5); (**B**) pH 3; (**C**) pH 8.5; (**D**) pH 9; (**E**) pH 10.5; (**F**) pH 11. Scale bar 10 μm.

**Figure 2:**
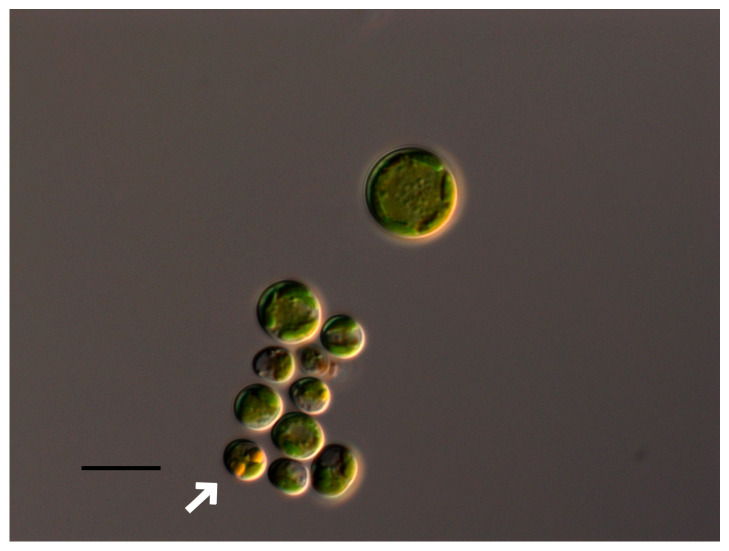
Oil-containing vacuoles in *Bracteacccus minor* cells (shown by white arrow). Scale bar 10 μm.

At pH 2–2.5, the complete discoloration and destruction of *Bracteacoccus minor* cells was observed. At pH 3, some of the cells lost their green color (Figure 1B). In the pH range of 4–10, the morphology of the algae cells was almost the same as that of the control variant (Figure 1A–D), and the appearance of large, pear-shaped cells was observed. It should be noted that such cells were often observed under normal conditions. At pH 3.5–5, some cells contained orange oil droplets (Figure 1B and Figure 2). At pH 10.5–11, discoloration of most of the cells was observed, and at pH 12–13.5, most *Bracteacoccus minor* cells were destroyed (Figure 1E,F).

**Figure 3:**
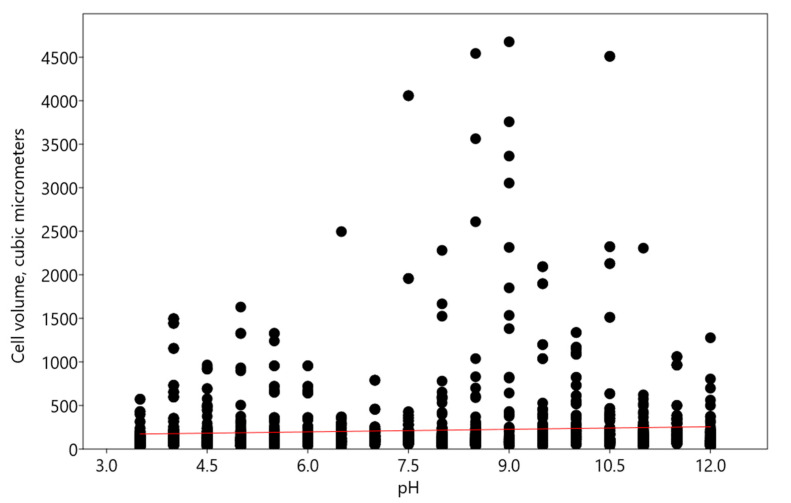
Influence of pH on cell volume of *Bracteacoccus minor*. Red line indicates the regression trend.

With the increase in pH concentrations, an increase in the volume of algal cells was observed according to a linear multivariate regression model, with pH as the independent variable, described by equation y = 9.8368x + 138.1 (Figure 3). These changes were significant despite the weakly expressed regression (R^2^: 0.004483; r: 0.066995; Wilks’ lambda: 0.9955; F: 9.209; p (regr): 0.002439).

### 2.2. Chlorococcum infusionum 

**Figure 4:**
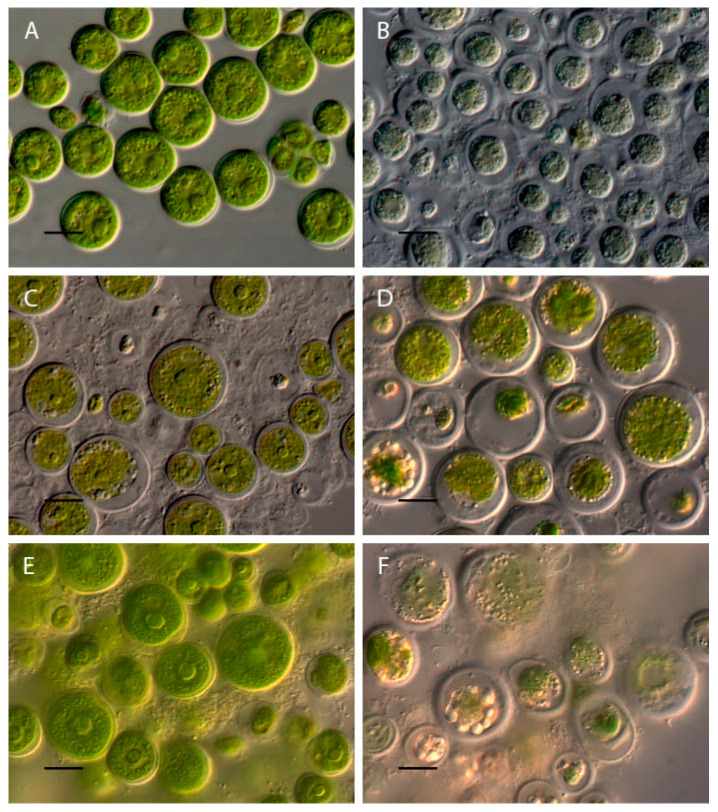
Influence of pH on *Chlorococcum infusionum* morphology. (**A**) Control variant (pH 6.5); (**B**) pH 2; (**C**) pH 3; (**D**) pH 3.5; (**E**) pH 10; (**F**) pH 12. Scale bar 10 μm.

In the acidic pH from 2 to 3.5, a loss of green color and the destruction of *Chlorococcum infusionum* cells were observed (Figure 4A,D). At pH 4–9.5, the algae retained the usual morphology, which did not differ from the morphology of algae in the control variant (Figure 4A). At pH 10–10.5, the destruction of some of the cells was observed. These cells showed a compressed protoplast, damage to the cell walls, and intensive granulation (Figure 4E). In the alkaline range of pH 11–13.5, all cells were discolored, with completely destroyed protoplasts (Figure 4F). 

**Figure 5:**
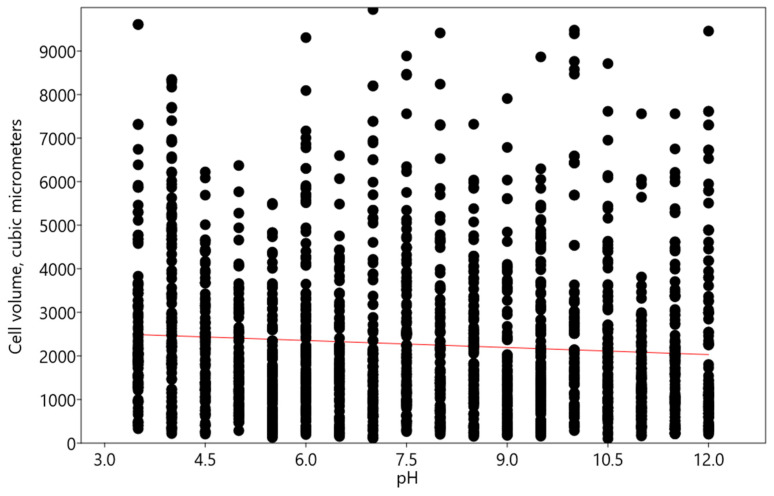
Influence of pH on cell volume of *Chlorococcum infusionum*. Red line indicates the regression trend.

With an increase in the pH, a reduction in the volume of *Chlorococcum infusionum* cells volume was detected. This change was described by a linear regression model y = −53.884x + 2677.9 (r: −0.07645; Wilks’ lambda: 0.9942; F: 11.13; p (regr): 0.0008664) (Figure 5). As in the experiments with *Bracteacoccus minor*, this effect on the cell size was very modest, even though the cell pigment and protoplast were significantly damaged. 

### 2.3. Chlorella vulgaris

**Figure 6:**
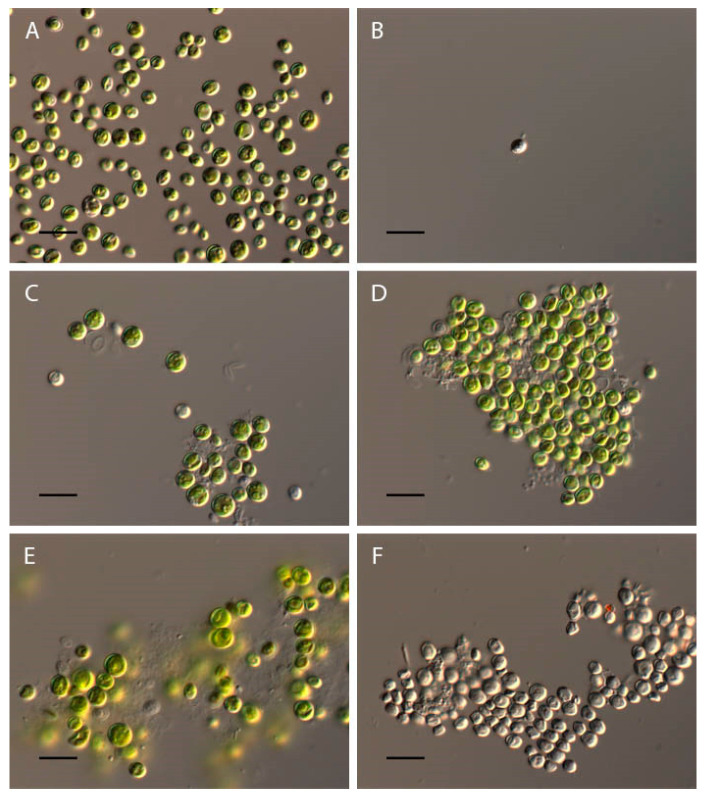
Influence of pH on *Chlorella vulgaris* morphology. (**A**) Control variant (pH 6.5); (**B**) pH 2; (**C**) pH 3.5; (**D**) pH 4; (**E**) pH 11.5; (**F**) pH 12. Scale bar 10 μm.

At pH 2–3, the complete discoloration of *Chlorella vulgaris* cells was observed (Figure 6A,B). At a pH of 3.5, about 30% of the cells lost their green color, but the remaining cells retained their usual morphology (Figure 6C). In the pH range from 4 to 11.5, the algae retained the morphological features of a green color, a cup-shaped chloroplast with the pyrenoid, and cell wall integrity (Figure 6D,E). At pH 12, the discoloration of all *Chlorella vulgaris* cells was observed (Figure 6F).

**Figure 7:**
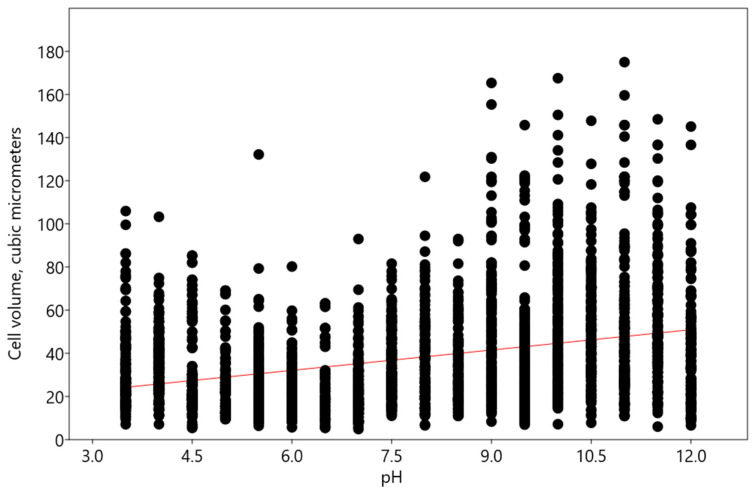
Influence of pH on cell volume of *Chlorella vulgaris.* Red line indicates the regression trend.

With a change in pH, an increase in the volume of *Chlorella vulgaris* cells was noted according to the linear multivariate regression model, with pH as the independent variable, described by the following equation: y = 3.1402x + 13.56 (R^2^: 0.1055; r: 0.32475; Wilks’ lambda: 0.8945; F: 305.7; p (regr): 8.51 × 10^−65^) (Figure 7).

Our results revealed a high resistance of *Chlorella vulgaris* to acid and alkaline pH levels, which is consistent with the information regarding the wide distribution of this species. 

### 2.4. Pseudococcomyxa simplex

**Figure 8:**
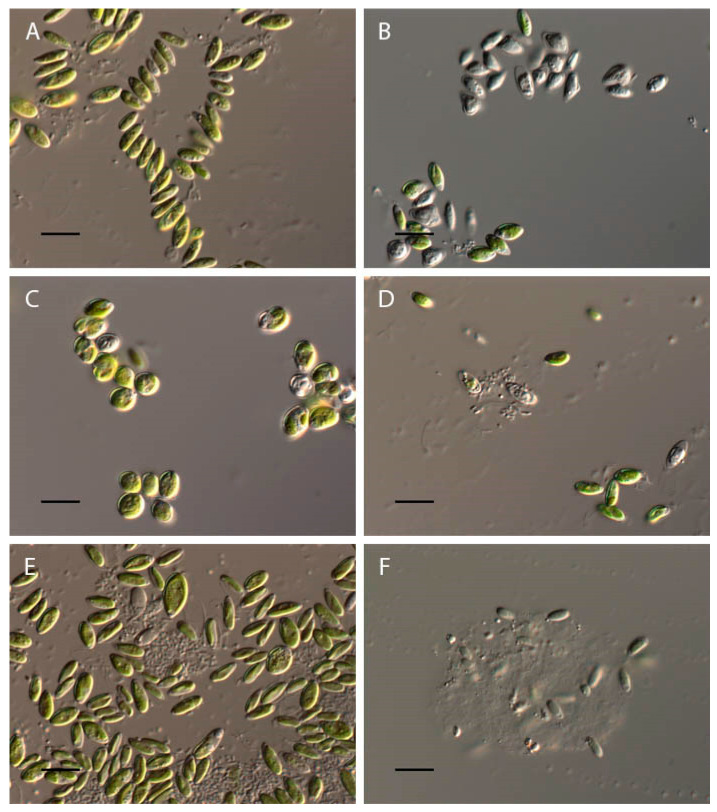
Influence of pH on *Pseudococcomyxa simplex* morphology. (**A**) control variant (pH 6.5); (**B**) pH 2; (**C**) pH 2.5; (**D**) pH 3.5; (**E**) pH 8.5; (**F**) pH 12. Scale bar 10 μm.

At pH 2, a loss of green color and “wrinkling” of *Pseudococcomyxa simplex* cells was noted (Figure 8A,B). At pH 2.5, about 50% of the cells were broadly ellipsoid in shape (Figure 8C). The appearance of larger cells was also recorded at a pH of 8.5–10.5 (Figure 8E). At a pH of 3.5, about 30% of the cells were discolored (Figure 8D). In the pH range of 4–11.5, the morphology of the algae was almost the same as that of the control variant (Figure 8A,E). At pH 12, complete discoloration of the algae cells was observed (Figure 8F).

**Figure 9:**
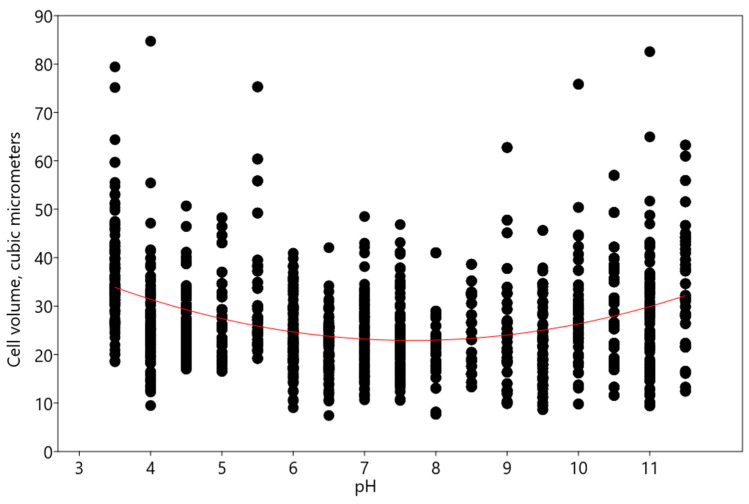
Influence of pH on cell volume of *Pseudococcomyxa simplex.* Red line indicates the regression trend.

With the change in pH, a change in the volume of algae cells, described by a polynomial regression model according to equation y= 0.6309x^2^ − 9.677x + 60.02, was detected. The reliability of this change was confirmed by the value of p (regr), which was significantly less than 0.05 (χ2: 1.719 × 10^5^; AIC: 4.572; R^2^: 0.10593; F: 105.62; p (regr): 4.4585 × 10^−44^) (Figure 9).

It should be noted that, at a low and high pH, *Pseudococcomyxa simplex* cells became similar to cells of *Avernensia* form [93,94], differing from *Pseudococcomyxa* by their more rounded cells. Perhaps *Avernensia* is the morphological form of *Pseudococcomyxa* in insufficient natural conditions. This fact should be taken into account during the identification of algae. 

### 2.5. Vischeria magna

**Figure 10:**
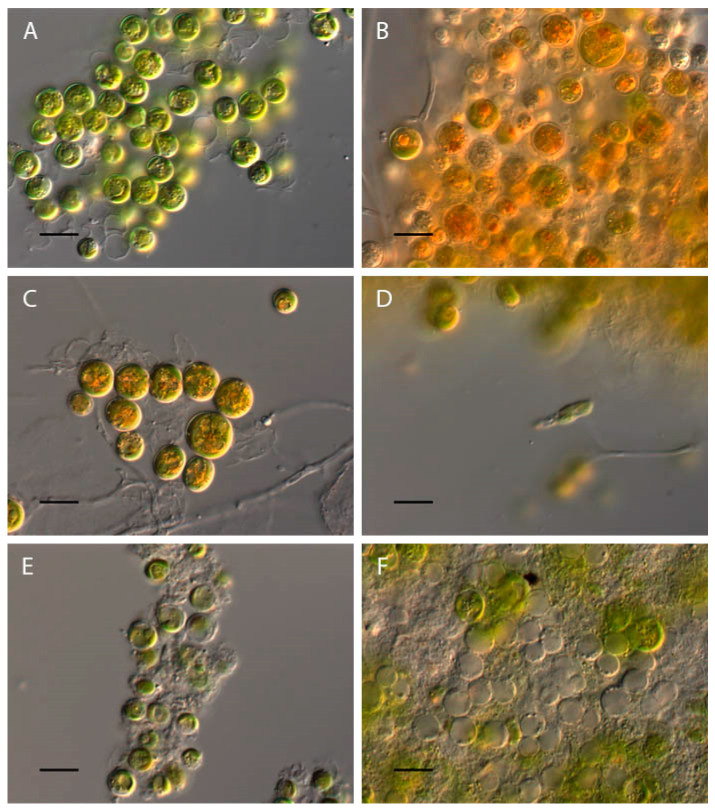
Influence of pH on *Vischeria magna* morphology. (**A**) Control variant (pH 6.5); (**B**) pH 2.5; (**C**) pH 3; (**D**) pH 3.5; (**E**) pH 11.5; (**F**) pH 12. Scale bar 10 μm.

At pH 2, complete discoloration of the *Vischeria magna* culture was observed. At pH 2.5, the algae cells were either completely discolored or bright orange (Figure 10A,B). Bright orange cells were also observed at a pH of 3 (Figure 10C). In the pH range of 3.5–11, the morphologies of most algae cells were the same as those of the control variant (Figure 10A,D), and zoospores were observed in this pH range, with the maximum at pH 3.5 (Figure 10D).

At high pH values from 11.5 to 12, 80% of *Vischeria magna* cells were discolored and destroyed (Figure 10E,F). At a pH level of 12.5–13.5, the culture was completely discolored.

**Figure 11:**
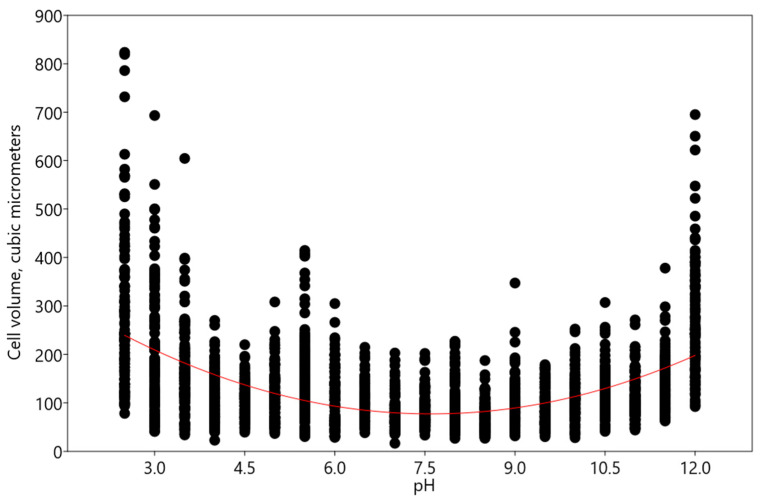
Influence of pH on cell volume of *Vischeria magna.* Red line indicates the regression trend.

The experiments revealed an increase in cell volume in the acidic and alkaline pH range, according to the polynomial regression model using equation y = 6.23x^2^ − 94.7x + 437.2 (χ2: 1.3735E07; AIC: 8.6522; R^2^: 0.28156; F: 471.47; p (regr): 1.7153 × 10^−173^) (Figure 11).

The study of pH’s influence on the morphology and cell volume of *Bracteacoccus minor*, *Chlorococcum infusionum*, *Chlorella vulgaris*, *Pseudococcomyxa simplex*, and *Vischeria magna* revealed that algae retain their morphological features at pH 3.5–4 and 9.5–11.5 (Table 1). Low and high pH levels mostly caused the discoloration and destruction of cells (Table 1), but specific reactions of individual species were also observed (Table 1). Low pH levels caused the appearance of broadly ellipsoid cells of *Pseudococcomyxa simplex* (Table 1 and Figure 8B), and orange granules in *Vischeria magna* cells (Table 1 and Figure 10B,C). 

## 3. Discussion

Our investigations revealed a high tolerance of the investigated algae species to low and high pH levels, which retained their basic morphological features in the pH range from 3.5–5 to 9.5–11.5 (Table 1). For some genera and species, these results correspond to previously published data. For example, *Chlorella pyrenoidosa* and *Chlorella ellipsoidea* can grow in media at pH levels 3.5 and 2, respectively, although the upper limit for their growth is about pH 10 [95]. 

The morphological disorders of algae cells at low and high pH levels are associated with the damaging effect of H^+^ and OH^−^ ions. Under very low pH conditions, cells experience stress because they must maintain a neutral pH level in the cytoplasm, since H^+^ ions continuously penetrate the plasma membranes [96]. Furthermore, low pH values can damage cell walls as a result of the weakening of hydrogen bonds in molecules, which can lead to an uncontrolled increase in cell size [97]. It has been established that acidification can influence metal bioavailability via the proton inhibition of facilitated metal uptake and by impacting membrane permeability [23]. It is known that, at high pH values, many elements necessary for organisms, such as Fe^2+^, Ca^2+^, Mg^2+^, and Zn^2+^, become insoluble and precipitate as carbonates, hydroxides, or phosphates. The concentration of hydrogen ions in the environment can affect the equilibrium of electric charges on the cell surface, increasing the total positive charge at low pH values or the total negative charge at high pH values [1]. Most likely, alkaliphilic algae, like other microorganisms, exclude hydroxyl groups, or, conversely, retain hydrogen ions [1].

Algae strains, which are resistant to extreme pH levels, have a great potential for use in biotechnology. pH is a critical parameter influencing the mass transfer rate of acidic gases like H_2_S and CO_2_ in conventional gas–liquid contactors [97,98]. At pH > 9 contaminants in the biogas die, while algal strains that are resistant to pH survive [99,100,101], The use of carbonated alkaline culture media in algal–bacterial photobioreactors supports long-term, effective biogas production [102]. pH is an important factor influencing the mechanism of algal bioremediation [103,104]. pH regulation can be used in the wastewater to remove emerging pollutants [104,105].

The resistance of algae to a wide range of pH values has been discussed in previous investigations [106,107,108,109]. *Euglena mutabilis*, a typical species in acidic environments, achieved maximal growth at pH 3.0–4.0, with a growth range of 2.0–9.0 pH [106]. For representatives of *Mougeotia*, in laboratory conditions the optimal pH was 8.0, but in nature, it was found to be pH 5.2 [107]. Chlamydomonas acidophila can grow at pH 1.5–7.0 [108]. The optimum pH values of three axenic strains of *Chloromonas tughillensis* were between 3.0 and 7.0, and of those of three non-axenic strains of *Chloromonas chenangoensis* were between pH 3.0 and 8.0 [109].

Some algae are tolerant to acidic environments [58,96,110,111,112]. These algae include the taxa investigated in our study. For example, in investigations of the algoflora of the Krušne hory Mts forests in northern Bohemia (Czech Republic), which are polluted by acid rain, *Bracteacoccus minor* and *Pseudococcomyxa simplex* were found in all investigated sites at pH 2.7–7.1. *Vischeria magna* and *Chlorella vulgaris* were also very frequent in the studied area [42]. *Pseudococcomyxa simplex* and representatives of genus *Chlorella—Chlorella saccharophila* and *Chlorella protococcoides*—were attributed to acidophilic and acidotolerant algae [97]. The strain *Coccomyxa melkonianii* SCCA 048 demonstrated phenotypic plasticity at different pH levels and active growth at pH = 4.0–8.0 [113]. In this study, at low pH levels (pH = 4), spherical-like cells were observed. We also detected similar morphological features at pH = 2.5. This phenomenon, discovered herein, once again demonstrates the morphological variability that is often observed in eukaryotic algae.

However, it should be noted that the floristic lists containing these data have not been confirmed by molecular–genetic methods. Therefore, information regarding the distribution of these species, which is difficult to determine, should be interpreted with caution.

There are also data regarding alkaliphilic and alcalitolerant algae. It has been found that eutrophic algae and cyanobacteria (*Gonatozygon monotaenium*, *Gloeocapsa* sp.) can grow at pH values above 9 [114]. Representatives of the genus *Klebsormidium*—*Klebsormidium acidophilum* and *Klebsormidium dissectum*—have been detected at pH 3.0 and pH 4.8–6.2, respectively [115]. High pH values (8–8.5), together with the high salinity, promoted the growth of the green filamentous alga *Chaetomorpha valida* [116].

Our studies also revealed a high resistance of *Chlorella vulgaris* to acidic and alkaline pH levels, which is consistent with information on the ubiquism of this species. As noted above, *Chlorella vulgaris* has been found in acidic soils [52]. However according to earlier investigations, the maximum growth of *Chlorella vulgaris* was observed at a pH of 6.31–6.84 [117]. The optimal pH for *Chlorella sorokiniana* DOE1412 is also approximately 6.0 for cell growth and lipid production [118].

The data regarding the resistance of *Chlorococcum infusionum* to different pH values are inconsistent. Some studies have revealed that *Chlorococcum infusionum* prefers neutral or slightly alkaline pH values [119]. There is also information about the resistance of *Chlorococcum* species to extreme pH conditions. Thus, in a study on the adaptation of the strain MS-1 *Chlorococcum* sp. to extreme alkalinity and salinity, the ability of algae to adapt and grow at a pH of higher than 12 was shown [120]. Simultaneously, *Chlorococcum infusionum* was found in very acidic habitats contaminated with heavy metals [121].

We found that changes in pH, especially at pH 3.5, caused mass zoospore germination in *Vischeria magna*. In the previous publication, spore germination in this species was observed at the pH levels at which it can grow [105]. It was noted that, most likely, akinetes could germinate at an acidic pH level due to the destruction of the akinete walls at a low pH [122]. Possibly, a similar mechanism caused the germination of the zoospores in *Vischeria magna* at low and high pH levels.

At a low pH (2.5–3) the orange oil granules were observed in *Vischeria magna*. This effect of enhanced lipid production could be used in biotechnology to increase oil production during the cultivation of representatives of *Eustigmatohyceae*. In previous publications, the impact of extreme levels of ecological factors on members of this family was assessed. For example, the influence of salt stress on a decrease in total productivity a very-long-chain fatty acid with high nutraceutical value productivity in an authentic *Vischeria punctata* strain, IPPAS H-242, was observed [123]. It was found that using nitrate–N, peptone-N sources, and NaCl allowed for oil production in *Vischeria* sp. WL1 strain to be increased [124].

We detected the appearance of oil granules in cells of *Bracteacoccus minor* at pH 3.5–5. According to previous data, under stress conditions, the representatives of the genus *Bracteacoccus* increase their oil production. For example, *Bracteacoccus bullatus* is suitable for the synthesis of oil with a high unsaturated fatty acid content at temperatures of 12–18 °C [125].

In previous investigations, a number of physiological mechanisms through which algae survive at low pH values have been described [26,97,108,112,126,127]. These mechanisms include maintaining a positive membrane potential [109], reducing the permeability of the plasma membrane to protons, and maintaining an active proton pump to preserve neutral cytosol [84,96,110]. The resistant plasma membrane also establishes the general tolerance of acidophilic and acidotolerant algae to heavy metals and toxic anions and prevents their entry into the algal cells [84,94,96]. In addition, it has been suggested that microalgae can undergo morphological adaptations to extremely low pH values [128].

It is generally believed that cells should have a low surface area to volume ratio (S/V) to reduce the stress caused by hydrogen cations penetrating through cell walls and membranes. A decrease in the S/V ratio can be achieved by increasing the volume of cells [26].

Our study reveals several models of changes in the biovolume of algal cells at extreme pH levels:

1. Minor changes according to the linear regression model—this type of reaction was characteristic of *Bracteacoccus minor* and *Chlorococcum infusionum*. It is necessary to note that desmid *Euastrum binale* also maintains a constant cell size when the pH changes [26]. It is possible that, for protection from extreme pH conditions, some species, such as *Bracteacoccus minor,* increased the oil content in their cells.
2. An increase in acidic and alkaline pH ranges according to the polynomial regression model—such changes were observed in *Pseudoccomixa simplex* and *Vischeria magna* cells. Perhaps these algae could utilize the regulation of biovolume under stress conditions of low and high pH levels. In addition, *Vischeria magna* increases the synthesis of oil-containing metabolites.
3. An increase in alkaline pH range according to the linear regression model was found in experiments with *Chlorella vulgaris*.

It is possible that the different reactions of algal biovolume at low and high pH levels could be explained by their taxonomic variability and related genetic, morphological, biochemical, physiological, and ecological peculiarities.

Algal strains, which are resistant to extreme pH conditions, have a great potential for use in biotechnology. The importance of studying the ecological resistance of algae was discussed in previous publications [29]. pH is a critical parameter influencing the mass transfer rate of acidic gases like H_2_S and CO_2_ in conventional gas–liquid contactors [98,99]. At pH > 9, the contaminants in the biogas die, while resistant-to-pH algae strains survive [100,101,102]. The use of carbonated alkaline culture media in algal–bacterial photobioreactors supports the idea of long-term effective biogas production [102]. Furthermore, the strains that are resistant to high and low pH levels can be used for industrial wastewater treatment, the pH of which ranges from 2 to 11 [129,130].

It should be emphasized that the studied strains are authentic (excluding *Vischeria magna*) and represented reference samples of these species. This allows us to consider their reactions to a change in pH as an example, and the reactions of other strains can later be compared with the reactions of investigated taxa. In addition, the studied strains are available in several major collections of algae and can be used in further physiological, biochemical, and biotechnological research. While different strains of *Chlorella* have already been used in biotechnology [131,132,133,134,135], the data concerning the strains investigated in our study are sporadic.

Thus, the investigated authentic and reference strains of *Chlorella vulgaris*, *Bracteacoccus minor*, *Pseudoccomyxa simplex*, *Chlorococcum infusionum*, and *Vischeria magna* were found to be resistant to extreme pH levels and to employ several physiological mechanisms for protection in low- and high pH conditions. These mechanisms include the changes in volume, the production of oil-containing substances, as well as the enhancement of zoospore production. The obtained results allow us to recommend the studied strains for broader use in biotechnology and nature protection.

## 4. Materials and Methods

### 4.1. Strains Cultivation and Methodic of Experiments

In this study, authentic strains or strains with correct identification were used. These strains were received from the Collection of Algae Culture of Taras Shevchenko Kiev National University (ACKU), which, in turn, received these strains from the Culture Collection of Algae at the University of Göttingen (SAG). The following strains were used in the experiments: *Chlorella vulgaris*—ACKU 531–06 (SAG (211–11b), *Bracteacoccus minor*—ACKU 506–06 (SAG 221–1), *Pseudoccomixa simplex*—ACKU 559–06 (SAG (216–9a), *Chlorococcum infusionum*—ACKU 539–06 (SAG 10.86), and UTEX (UTEX 2351 *Eustigmatos magna* = *Vischeria magna*).

Strains were cultivated in the liquid Bold media [136] in a 250 mL flask at 20–23 °C at 12:12 h light–dark cycle with an illumination of 40 μmol/m^2^ per second, using 18W cool fluorescent tubes (Philips TLD18W/33, Philips Lighting Poland S.A., Pila, Poland), for 14 days. For the experiments, algal cultures containing 1 × 10^8^ cells algae cells per 1 mL were used.

Prior to the experiments, the pH meter electrodes were calibrated using standard buffer solutions (pH = 3.56, pH = 6.86, and pH = 9.18). In the experiments, pH levels from 2 to 13.5, with an interval of 0.5, were studied. The liquid Bold media with a pH of 6.5 acted as the control. The different pH values were achieved by adding 0.1 molar solutions of NaOH or HCl, which were added into the Bold media. The pH of the media was measured using a pH meter Multitest IPL-311 with an ESC electrode (No. 1060317) (Semiko, Novosibirsk, Russia), and 1 mL of algae suspension was transferred into the tubes containing 15 mL of solutions of different pH concentrations. The tubes were incubated for 7 days under the conditions described above.

For each pH concentration, 5 tubes were examined. From each tube, 10–15 micrographs, containing at least 20 cells each, were taken. Then, the dimensions of all cells from several micrographs were determined in such a way that, for each concentration, measurements were taken of the length, width, and other dimensions of at least 100 algal cells.

For morphological observation of the algae, an Axio Imager A2 microscope (Carl Zeiss, Oberkochen, Germany), equipped with Nomarski DIC optics, was used. Algae micrographs were taken using an Axio Cam MRC (Carl Zeiss, Oberkochen, Germany) camera with magnification ×1000 in oil immersion, and AxioVision 4.9.1 software was employed. The dimensions of algae cells were also determined using AxioVision 4.9.1 software.

### 4.2. Statistical Analysis

Statistical processing of the results was carried out using regression analysis [137]. To calculate the biological volume of the algae (V), the formulae of geometric shapes that most accurately corresponded to the shapes of their bodies were used [78]. The volume of globular cells of *Chlorella vulgaris*, *Bracteacoccus minor, Chlorococcum infusionum*, and *Vischeria magna* were calculated by sphere volume using the formula:

V = π/6 a^3^ (1)

where a is the diameter of the sphere [78].

It should be noted that the young cells of *Chlorella vulgaris* and *Chlorococcum infu-sionum* have an elongated shape. Therefore, to calculate the cell volumes of these species and *Pseudococcomyxa simplex* cells, the ellipsoid formula was used:

V = π/6 b^2^ a (2)

where a is the length of the major axis and b is the length of the minor axis [78].

During the analysis of the pH value’s influence on the volume of *Chlorella vulgaris* and *Chlorococcum infusionum* cells, the volumes of both spherical and ellipsoid cells were taken into account.

Statistical processing of the results was carried out using the Past 2.14 software [138]. The morphological variations related to the changing pH values were analyzed. For this purpose, the most suitable regression model was chosen.

## Figures and Tables

**Table 1:** The limits of pH and morphological disturbances at a low and high pH.

Taxa	pH Limits	Morphological and Physiological Features Change at pH < 4	Morphological and Physiological Features at pH > 10
*Bracteacoccus* *minor*	4–10	Complete discoloration and destruction of cells; the appearance of orange granules	Discoloration and destruction of cells
*Chlorococcum infusionum*	4–9.5	Discoloration and destruction of cells; the appearance of large granules in cytoplasm	Discoloration and destruction of cells, appearance of large granules in cytoplasm
*Chlorella vulgaris*	4–11.5	Discoloration of cells	Discoloration of cells
*Pseudococcomyxa* *simplex*	4–11.5	Discoloration and “wrinkling” of cells; appearance of almost round cells	Discoloration of cells
*Vischeria* *magna*	3.5–11	Discoloration and appearance of the orange granules in cells; maximal zoospores production at pH 3.5; increase in cell volume	Discoloration of cells

## Data Availability

Data are contained within the article.
